# Assessing Airflow Limitation among Smokers in a Primary Care Setting

**DOI:** 10.21315/mjms2018.25.3.8

**Published:** 2018-06-28

**Authors:** Chean Kooi Yau, Fairuz Fadzilah Rahim, Chin Jiunn Sheng, Choi Xin Ling, Liew Kah Weng, Tan Chia Chia, Tan Kean Chye, Ooi Siew Ting, Tan Hong Jin, Irfhan Ali Hyder Ali

**Affiliations:** 1 Department of Family Medicine, Penang Medical College, 4, Jalan Sepoy Lines, 10450 Pulau Pinang, Malaysia; 2 Department of Public Health, Penang Medical College, 4, Jalan Sepoy Lines, 10450 Pulau Pinang, Malaysia; 3 Trinity College Dublin, School of Medicine, Dublin 2, Ireland; 4 Department of Primary Care, Penang General Hospital, Jalan Residensi, 10450 Pulau Pinang, Malaysia; 5 Department of Respiratory Medicine, Penang General Hospital, Jalan Residensi, 10450 Pulau Pinang, Malaysia

**Keywords:** airflow limitation, COPD, pocket spirometry, primary care, quit smoking, smokers

## Abstract

**Background:**

Many smokers have undiagnosed chronic obstructive pulmonary disease (COPD), and yet screening for COPD is not recommended. Smokers who know that they have airflow limitation are more likely to quit smoking. This study aims to identify the prevalence and predictors of airflow limitation among smokers in primary care.

**Methods:**

Current smokers ≥ 40 years old who were asymptomatic clinic attendees in a primary care setting were recruited consecutively for two months. We used a two-step strategy. Step 1: participants filled in a questionnaire. Step 2: Assessment of airflow limitation using a pocket spirometer. Multiple logistic regression was utilised to determine the best risk predictors for airflow limitation.

**Results:**

Three hundred participants were recruited. Mean age was 58.35 (SD 10.30) years old and mean smoking history was 34.56 pack-years (SD 25.23). One in two smokers were found to have airflow limitation; the predictors were Indian ethnicity, prolonged smoking pack-year history and Lung Function Questionnaire score ≤ 18. Readiness to quit smoking and the awareness of COPD were low.

**Conclusions:**

The high prevalence of airflow limitation and low readiness to quit smoking imply urgency with helping smokers to quit smoking. Identifying airflow limitation as an additional motivator for smoking cessation intervention may be considered. A two-step case-finding method is potentially feasible.

## Introduction

Chronic obstructive pulmonary disease (COPD) is a major cause of mortality with increasing incidence. The World Health Organisation predicts that COPD will become the third major cause of global death by 2030 (1). COPD is not curable but it is treatable and preventable (1–3). Unfortunately, it is often underdiagnosed (3–5) and therefore untreated as a result (6). In Malaysia, the prevalence of current smokers is 22.8%, and it is estimated that nearly five million Malaysians aged 15 years and above smoke (7). The published prevalence of COPD in Malaysia is comparatively low at around 3.4% to 6.5% (8).

Screening for COPD remains controversial. The US Preventive Services Task Force (USPSTF) recommended against screening for COPD in asymptomatic adults, justified by the lack of evidence in net patient benefit and cost effectiveness in screening (9). However, some experts have supported early detection of COPD before patients recognise their symptoms (3, 10, 11), and the current findings favour targeted case finding (3, 11, 12) rather than population screening. In particular, active case finding is more cost-effective when compared with opportunistic case finding (5). Nevertheless, the best approach for such COPD case-finding strategies is yet to be established.

The current understanding is that a firm diagnosis of COPD is not necessary in smokers (3, 9). In addition, we also know that quitting smoking is the only option to prevent COPD (2) and smokers who know they have airflow limitation are more motivated to quit smoking (13–15).

This study aimed to determine the prevalence and predictive factors for airflow limitation among current smokers in primary care. We utilised a two-step case finding strategy by using a screening questionnaire and an affordable pocket spirometer to detect airflow limitation, targeting current smokers.

## Methods

### Study Design and Participants

This is a cross-sectional study registered and approved by Medical Research Ethics Committee of the Ministry of Health Malaysia (NMRR ID: 31737). It was conducted at the primary care clinic (outpatient clinic) of Penang General Hospital. In August to October 2016, all patients ≥ 40 years old who attended the primary care clinic were screened consecutively. Eligible participants were current smokers who had no known lung disease or acute respiratory problem and were not pregnant. In addition, subjects must have been able to perform all study-related protocols, including a technically acceptable pulmonary function test. Current smokers were defined as participants who smoked at least 100 cigarettes, including rolled cigarettes, pipes and cigars in their lifetime and who, at the time of study, smoked either every day or on some days. (16)

### Survey Instruments and Administration

**Figure 1:**
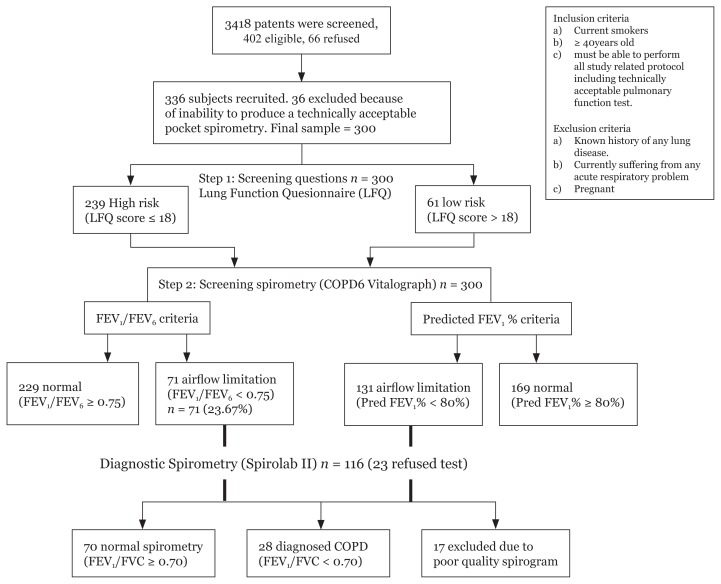
Flow chart of participants in the study

Written consent was obtained from all participants. A two-step case-finding strategy was used. (Figure 1)

#### Step 1: Questionnaire

A face-to-face interview was carried out to obtain relevant information from patients after a clear explanation of each question in the questionnaire. The questionnaire consisted of the following:

- Basic demography: age, gender, ethnicity, educational level, income, weight and height.
- Smoking history: duration of smoking, number of cigarettes per day and if they had made any previous attempts to quit.
- Fagerström test (17)
  This is a standard instrument designed to assess the intensity of physical addiction to nicotine. The questionnaire consists of six items which evaluate the quantity of cigarette consumption, the compulsion to use and dependence. The items are summed to yield a total score of 0–10. The higher the total score, the more intense the participant’s nicotine dependence (17).
- Awareness of chronic obstructive pulmonary disease (COPD)
  Subjects were first asked if they had heard of COPD before; if the answer given was “no”, the researchers would then describe COPD with a standardised phrase explaining COPD as “narrowing of the airways commonly caused by smoking.”
- “Readiness to quit smoking” using Prochaska and DiClemente’s Stages of Change model (18)
  This model is a component of the transtheoretical model of behaviour change (TTM). The stages are classified as: pre-contemplation (currently smoking and not planning to give up), contemplation (currently smoking with a desire to give up smoking but not in the next month), preparation for action (currently smoking and planning a quit attempt in the next month), action (had smoked in the past year but were no longer smoking), maintenance (had smoked sometime in the past but not in the previous year). The “action” and “maintenance” stages were not applicable in this study because all subjects were current smokers.
- Lung function questionnaire (LFQ) (19, 20)
  This is a validated (20) questionnaire consisting of questions which captured information such as frequency of chesty cough, wheezing and dyspnoea during physical activity, years of smoking and age of the patients; a five-point Likert scale was used. Hence, the total score could range from 5 to 25. A score 18 or less suggests an increased risk of COPD (19).

### Step 2: Lung function assessment

Upon completion of the questionnaire, participants would then proceed to the lung function assessment as follows:

- Assessment of airflow limitation using a pocket spirometer, COPD-6 (Model 4000 Vitalograph, Ennis, Ireland)
  The Vitalograph COPD-6 measures forced expiratory volume in first seconds (FEV_1_), forced expiratory volume in first six seconds (FEV_6_), FEV_1_% and FEV_1_/FEV_6_. It is an effective and validated option to detect airflow limitation and to diagnose COPD (21) in primary care. To ensure the validity of the study, all six investigators of the team underwent training using an online training video (22) and all pocket spirometry devices were calibrated before data collection. In addition, pilot testing involving 20 participants was carried out to ensure competency and consistency amongst the investigators in mastering the study instrument.
  Explanations and demonstrations were done for every subject before they attempted. A forced expiration for at least six seconds with minimal air leak was considered a good quality blow, guided by the blow quality indicator on the device. If an exclamation mark (!) appeared, indicating the blow was not of good quality, the participants would be required to attempt again until a good quality blow was obtained. Subsequently, the best readings of three valid attempts were recorded. Airflow limitation was defined as FEV_1_/FEV_6_ ratio< 0.75 or FEV_1_ < 80% predicted (23).
- Diagnostic spirometry using Spirolab II
  Participants who were found to have airflow limitation were then referred to the respiratory unit at Penang General Hospital for diagnostic spirometry. COPD was diagnosed when FEV_1_/FVC < 0.70 (9).

### Statistical Analysis

All data were entered into Microsoft Excel. Data cleaning, exploration and analysis were performed using Stata/SE Version 13 (24). Means [standard deviation (SD)] were reported for continuous variables. Frequencies and proportions were reported if data were categorical variables. The logistic regression analyses were conducted to predict the risk associated with the positive pocket spirometry outcome. Simple logistic regression assessed the univariable effect of possible predictors to the outcome. Variable selection for consideration of multiple logistic regression model was based on the principles of a) fit [variables that showed statistically significant bivariate association with the outcome at *P* < 0.25 (25)] b) parsimony, and c) biological plausibility [variables with a potential clinically significant association with the outcome, such as gender (26, 27) and marital status (27)]. The final model produced adjusted odds ratios [95% confidence interval (CI)]. The fitness of the model was also assessed.

## Results

### Characteristics of the Participants

Of the 402 participants who were eligible, 66 of them refused. The main reasons for refusal were time constraint and shyness. A further 36 participants were excluded because of inability to perform technically acceptable pocket spirometry. A final sample of 300 was recruited (Figure 1).

The majority (95.33%) of the participants were male and only 4.67% were female. The mean age was 58.35 (SD 10.30) years old. The average body mass index (BMI) was 24.55 (SD 5.11). More than half of smokers had monthly income less than RM1000 (54.67%).

The mean smoking history was 34.56 (SD 25.23) pack-years (Table 1). Nearly two-thirds of our participants had previously attempted to quit (63.00%). The Fageström test showed more than half of the participants had very low addiction (33.33%) and low addiction (26.00%). About one quarter of the participants had high (16.67%) and very high addiction (10.33%). In the Prochaska and DiClemente’s Stages of Change model for the intention to quit smoking, only one out of five patients were in the preparation for action stage (19.33%). The majority reported being at the pre-contemplation stage (68.33%) and contemplation stage (12.33%). Awareness of COPD was low with 71.33% of participants having never heard of COPD before. 79.67% were identified as being at risk of COPD with an LFQ score ≤ 18.

### Prevalence and Predictors of Airflow Limitation

When screened with the pocket spirometer, 139 participants (46.33%) were found to have airflow limitation. Of these, 23 refused diagnostic spirometry and thus 116 proceeded to undergo diagnostic spirometry. Subsequently, 28 participants (9.3%) were diagnosed with COPD (Figure 1).

Using multiple logistic regression, the analysis was carried out by backward stepwise and forward selection, then the enter method was performed for the chosen and clinically significant variables. Models were compared based on R^2^ value (the percentage of variance in the outcome explained by the model), level of significance, percentage of outcome categories correctly explained and number of significant predictors in the model. The backward stepwise method provided the best prediction model. We identified three predictors significantly associated with screening of airflow limitation by pocket spirometry (Table 2).

- Ethnicity
  Indian participants were more likely to have airflow limitation when compared with Malays (adjusted OR= 0.28 95% CI: 0.12–0.67) and Chinese (adjusted OR= 0.23 95% CI: 0.10–0.52). Other ethnicities were omitted due to the very small number of participants.
- Smoking history in pack-years
  A prolonged smoking pack year history was significantly associated with airflow limitation. The risk of having airflow limitation increased by 2% for each one unit increase in smoking pack year history (adjusted OR= 1.02 95% CI: 1.01–1.03).
- Lung Function Questionnaire (LFQ) score
  Participants who had a LFQ score ≤ 18 were two times more likely to have airflow limitation. (adjusted OR= 2.19 95% CI: 1.12–4.27).

## Discussion

To the best of our knowledge, this is the first study to perform a risk assessment for COPD on current smokers in Malaysia. We found a high prevalence of airflow limitation. The predictors of airflow limitation were Indian ethnicity, LFQ ≤ 18 and a long smoking history in pack-years. In addition, the awareness of COPD and the intention to quit smoking were both low among current smokers. Such findings should alert us to look out for more smokers who are at a high risk of COPD and subsequently to raise their awareness and to motivate them to quit smoking.

Compared with a local study published by Ching and colleagues, (28) the prevalence of airflow limitation in our participants was higher (46.3% versus 10.6%). It is important to note that we recruited from only current smokers and not the general population. Furthermore, our study population included heavier smokers (34.56 versus 20.4 pack-years) and was older (58 versus 54 years old). These are known risk factors for COPD.

A lifelong smoker will have at least one in two chances of developing COPD (2). Indeed, one in two current smokers in our study population had undiagnosed airflow limitation detected using a pocket spirometer. The two main reasons for the underdiagnosis of COPD have been reported as the underutilisation of spirometry by doctors and the ignorance of patients regarding the signs, symptoms and risk factors (3). Awareness of COPD among the smokers in this study was low, with 71.3% having not heard of COPD before. As a result, individuals at risk may not seek attention for diagnosis and treatment. Many smokers attribute their respiratory symptoms to part of the aging process (29) or a lack of fitness. Some regard their condition as a natural side effect of tobacco cigarettes smoking instead of COPD itself. The Global Initiative for Chronic Obstructive Lung Disease (GOLD) (30) states, “A diagnosis of COPD should be considered in any patient who has dyspnoea, chronic cough or sputum production and a history of exposure to risk factors”. In reality, the presentation of COPD symptoms to family doctors is poor. The current recommendation to not screen asymptomatic patients should therefore be interpreted with caution in those who are not truly asymptomatic but are at higher risk for COPD (31). Primary care doctors could therefore play a pioneering role in addressing this problem by first screening patients for COPD symptoms.

Smoking cessation is the only effective “treatment” for those at risk and undiagnosed COPD patients. It decreases risk of developing COPD by about half (2). All smokers should of course be counselled to stop smoking, but the reality is that smoking cessation is challenging in that it has low yield in terms of the quit rate. Studies have shown that patients who were told that they have airflow limitation and who undergo more intensive smoking cessation are more likely to quit (14). Even worrying about COPD itself can increase a smoker’s motivational level to stop smoking (15, 32). Programmes involving the detection of new COPD cases could lead to higher smoking cessation rates (14). Similarly, a qualitative study reported that the majority of smokers agreed that measuring and confronting them with their lung function was justifiable in helping with attempts to quit (15, 33).

In Malaysia, diagnostic spirometry testing for COPD is available mainly in tertiary settings. The cost of a pocket spirometer (Vitalograph) is less than 1/10 the cost of a diagnostic spirometer (Spirolab). It is sufficiently affordable for all general practitioners to detect potential COPD patients. SEARCH 1, a prospective cluster of randomised trials, used the COPD-population screener (COPD-PS) questionnaire, which contained similar questions as the LFQ used in this study. Together with a pocket spirometer, this increased COPD diagnostic yield by 1.16% in 8 weeks (34). Thus, we believe that the outcome of this two-step screening (Figure 1) has a potential role as a motivational tool for smoking cessation. Similarly, primary care doctors may be more likely to counsel patients for additional smoking cessation therapies based on spirometric findings.

A new finding in this study is that Indian ethnicity is a risk factor for COPD when compared with Malays and Chinese. Studies have suggested that COPD risk varies by ethnicity. For example, the white population has a higher prevalence of COPD when compared with South Asian and black patients. (35) Two other predictors for airflow limitation were smokers with an LFQ score ≤ 18 and a prolonged smoking history in pack-years. The elements in these predictors are consistent with previous research evidence (36).

The limitations of this study should be noted. Post-bronchodilator spirometry was not performed to confirm the diagnosis of COPD. In addition, not all the participants in this study underwent diagnostic spirometry; only those with airflow limitation did. This potentially missed some underdiagnosed cases. Therefore, the prevalence of COPD in this study should be interpreted with caution. However, it be noted that smokers with known respiratory problems, especially asthma, were excluded in this study and hence cases of reversible airflow obstruction were therefore minimised. Lastly, the performance of our multiple logistic regression model was relatively low. Perhaps there are other predictors outside the scope of this study to consider.

This study was done in a primary care clinic within a hospital setting. There is a need to obtain prevalence from other populations such as community clinics to triangulate the findings of this study. Further studies using the same method, preferably at multiple cross-sectional sites, is recommended. Finally, cohort studies to determine the outcomes of intervention related to COPD risk assessment would be useful.

## Conclusion

Although it has been well-established that screening for COPD is not recommended as long as patients are asymptomatic, patients do not generally present themselves to their doctors with “symptoms”, and therefore COPD is underdiagnosed. In this sample, about half of the smokers suffered from airflow limitation. In addition, their awareness of COPD and intention to quit smoking were low. Therefore, identifying airflow limitation as a motivator to quit smoking is justified for COPD prevention.

## Figures and Tables

**Table 1:** Univariable analysis of socio-demographic characteristics, physical measurements and smoking on pocket spirometry outcome, assessed by simple logistic regression (*N* = 300)

Factors	*n* (%)			Crude Odds Ratio (95% CI)	*P*-value

	Total	Airflow limitation (*n* = 139)	No airflow limitation (*n* = 161)		
Age (years)^a^	58.35 (10.30)	60.24 (10.67)	56.73 (9.71)	1.03 (1.01, 1.06)	0.004
Age group (years)					
Middle aged	165 (55.00)	65 (46.76)	100 (62.11)	1	
Elderly	135 (45.00)	74 (53.24)	61 (37.89)	1.87 (0.48, 0.89)	0.008
Gender					
Male	286 (95.33)	132 (94.96)	154 (95.65)	1	
Female	14 (4.67)	7 (5.04)	7 (4.35)	1.17 (0.40, 3.41)	0.778
Ethnicity					
Indian	37 (12.33)	25 (17.99)	12 (7.45)	1	
Malay	75 (25.00)	31 (22.30)	44 (27.33)	0.34 (0.15, 0.77)	0.010
Chinese	186 (62.00)	83 (59.71)	103 (63.98)	0.39 (0.18, 0.82)	0.013
Others	2 (0.67)	0 (0.00)	2 (1.24)	*	*
Marital status					
Single	65 (21.67)	26 (18.71)	39 (24.22)	1	
Married	207 (69.00)	98 (70.50)	109 (67.70)	1.35 (0.77, 2.38)	0.301
Divorced	21 (7.00)	11 (7.91)	10 (6.21)	1.65 (0.61, 4.44)	0.321
Widowed	7 (2.33)	4 (2.88)	3 (1.86)	2.00 (0.41, 9.68)	0.389
Income					
< RM 1000	164 (54.67)	85 (61.15)	79 (49.07)	1	
RM 1001–RM 5000	133 (44.33)	53 (38.13)	80 (49.69)	0.62 (0.39, 0.98)	0.040
RM 5001–RM 10000	3 (1.00)	1 (0.72)	2 (1.24)	0.46 (0.04, 5.23)	0.535
Level of education					
No education	9 (3.00)	6 (4.32)	3 (1.86)	1	
Primary	96 (32.00)	47 (33.81)	49 (30.43)	0.48 (0.11, 2.03)	0.318
Secondary	175 (58.33)	79 (56.83)	96 (59.63)	0.41 (0.10, 1.70)	0.219
Tertiary	20 (6.67)	7 (5.04)	13 (8.07)	0.27 (0.05, 1.42)	0.122
BMI (kg/m^2^) a	24.55 (5.11)	24.47 (5.95)	24.63 (4.27)	0.99 (0.95, 1.04)	0.789
BMI category					
Underweight	22 (7.33)	13 (9.35)	9 (5.59)	1	
Normal	149 (49.67)	68 (48.92)	81 (50.31)	1.72 (0.69, 4.27)	0.242
Overweight	98 (32.67)	41 (29.50)	57 (35.40)	0.86 (0.51, 1.43)	0.556
Obese	31 (10.33)	17 (12.23)	14 (8.70)	1.45 (0.66, 3.15)	0.352
Awareness of COPD					
Yes	86 (28.67)	31 (22.30)	55 (34.16)	1	
No	214 (71.33)	108 (77.70)	106 (65.84)	1.81 (1.08, 3.03)	0.024
Smoking History					
Ever tried to quit smoking					
Yes	189 (63.00)	82 (58.99)	107 (66.46)	1	
No	111 (37.00)	57 (41.01)	54 (33.54)	1.38 (0.86, 2.20)	0.182
Smoking duration (years) a	38.17 (12.96)	40.35 (13.39)	36.29 (12.31)	1.03 (1.01, 1.04)	0.007
Numbers of stick per day a	17.49 (10.80)	18.99 (10.40)	16.19 (10.99)	1.02 (1.00, 1.05)	0.026
Pack-year smoking a	34.56 (25.23)	40.12 (24.35)	34.12 (25.95)	1.02 (1.01, 1.03)	0.001
Readiness to quit smoking					
Preparation for action	58 (19.33)	32 (19.88)	26 (18.71)	1	
Contemplating	37 (12.33)	20 (12.42)	17 (12.23)	1.05 (0.46, 2.39)	0.915
Pre-contemplating	205 (68.33)	109 (67.70)	96 (69.06)	1.08 (0.60, 1.94)	0.787
LFQ score					
More than 18	61 (20.33)	17 (12.23)	44 (27.33)	1	
Less and equal 18	239 (79.67)	122 (87.77)	117 (72.67)	2.70 (1.46, 4.99)	0.002

a Mean (SD)

**Table 2:** Predictors associated with airflow limitation, assessed by multiple logistic regression* (*N* = 298)

Factors	Crude OR (95% CI)	Adjusted OR* (95% CI)	*P*-value
Ethnicity			
Indian	1	1	
Malay	0.34 (0.15, 0.77)	0.28 (0.12, 0.67)	0.004
Chinese	0.39 (0.18, 0.82)	0.23 (0.10, 0.52)	< 0.001
Pack year smoking	1.02 (1.01, 1.03)	1.02 (1.01, 1.03)	0.002
LFQ score			
More than 18	1	1	
Equal or less than 18	2.70 (1.46, 4.99)	2.19 (1.12, 4.27)	0.021

* Backward stepwise logistic regression was applied. Multicollinearity and interaction were not found.

* Regression model was satisfactory fit: Hosmer-Lemeshow (Chi^2^(df) = 13.66(8); *P*-value = 0.091; Overall correctly classified percentage = 63.8%; Area under the ROC Curve = 67.2%)

OR: odds ratio; CI: confidence interval; LFQ: Lung Function Questionnaire

## Abbreviations

FEV_6_: forced expiratory volume in first six seconds
FVC: forced vital capacity
OR: odds ratio.
